# Catalytic and structural comparisons of linoleate dioxygenases and their cytochrome P450 companions with enzymes of the cyclooxygenase cascade

**DOI:** 10.1016/j.jbc.2026.111263

**Published:** 2026-02-06

**Authors:** Ernst H. Oliw

**Affiliations:** Department of Pharmaceutical Biosciences, Uppsala University, Uppsala, Sweden

**Keywords:** AlphaFold 2, computed structure models, cytochrome P450, mutagenesis site-specific, oxygenation mechanism, oxylipin biosynthesis, peroxidase

## Abstract

Prostaglandin H_2_ (PGH_2_) is formed from arachidonic acid by cyclooxygenases (COXs) and metabolized by thromboxane (TXS) and prostacyclin synthase (PGIS), two self-sufficient cytochromes P450 (CYP). The related fungal linoleic acid (LA) biosynthetic route is catalyzed by diheme proteins of five dioxygenases (DOXs) fused to three akin CYP subfamilies of allene oxide (AOS), linoleate diol (LDS), and epoxy alcohol (EAS) synthases. AlphaFold2 predicted the 3D structures of the DOX-CYP domains with very high confidence. Superposition with the COX:LA enzyme complex indicated that the protein fold of central α-helices and the motifs of the substrate recognition sites (SRSs) were conserved, which suggest evolution from an ancient peroxidase precursor. TXS, PGIS, and AOS catalyze homolytic scissions of oxygen–oxygen bonds and LDS/EAS heterolytic scissions. The SRS4 of LDS and EAS predicted an Asn residue at close distal axial position of the heme thiolate iron in analogy with PGIS and plant AOS, but a nonpolar in TXS and 8*S*/9*S*-AOS, and a polar (Thr) in 8*R*/9*R*-AOS. Replacements of amide residues in SRS4 of LDSs shifted the position of intramolecular hydroxylation of 8*R*-hydroperoxy-LA and the heterolytic scission to towards homolytic. The self-sufficient CYP may catalyze homolytic and heterolytic cleavage of hydroperoxides and the endoperoxide of prostaglandin H_2_ by different mechanisms, but the presentation of the oxygen–oxygen bonds to the metal centers might be crucial. The AF2 models illustrate the structural, catalytical, and evolutionary relationships between COX and microbiological DOX with CYP companions in unprecedented details, which reveal multiple amino acids of potential catalytic significance for future research.

## Introduction

Eicosanoids and octadecanoids are oxylipins, formed by oxidation of unsaturated fatty acids in man and lower organisms (1, 2, 3, 4, 5, 6, 7). Eicosanoids are produced from arachidonic acid (AA) and play pivotal roles in pain, inflammation, asthma, cardiovascular disease, and human reproduction (6, 8, 9). Plants and fungi produce octadecanoids, which are formed from oleic acid (OA), linoleic acid (LA), or α-linolenic acid (ALA), and participate in reproduction, development, and host–pathogen interactions (4, 5, 7, 10, 11, 12, 13, 14, 15, 16). Biosynthesis of oxylipins from these unsaturated fatty acids can be initiated by two different families of metal dioxygenases (DOXs).

Lipoxygenases (LOXs) are non-heme Fe- or Mn-containing enzymes, which oxidize the *cis-cis* 1,4-pentadiene of unsaturated fatty acids by hydrogen abstraction at C3 and O_2_ insertion at C1, C3, or C5 (17, 18, 19, 20, 21). The second family consists of two isozymes, cyclooxygenase-1 (COX-1) and COX-2, and related heme DOXs of fungi, plants, and cyanobacteria. COX oxidizes AA to prostaglandin H_2_ (PGH_2_) by abstraction of the 13*S* hydrogen by a tyrosyl radical, insertion of O_2_ at C11, rearrangement to a 9,11-endoperoxide and a five membered carbon ring, and addition of O_2_ at C15 (1, 2, 3, 22, 23). The tyrosyl radical is formed by oxidation of the heme cofactor by peroxides to a ferryl intermediate (compound I), which then oxidizes the nearby Tyr385 (24). The fungal DOX can also be oxidized in this way to generate a tyrosyl radical, which abstracts hydrogens at allylic or bis-allylic carbons for successive insertion of O_2_ and formation of hydroperoxides (5, 25, 26, 27). The α-DOX of plants and cyanobacteria oxidize C2 of fatty acids to hydroperoxides by the same mechanism (28, 29, 30, 31).

Metabolites of AA, which are produced by LOX and COX, can be further transformed by leukotriene and prostaglandin E synthases and by many other enzymes (1, 2, 3, 6, 7). PGH_2_ can, for example, be isomerized by thromboxane synthase (TXS; CYP5A1) of platelets and prostacyclin synthase (PGIS; CYP8A1) of the vasculature, which produce two physiological mediators with imbalance in cardiovascular disease (1, 8, 9). These two CYP do not require an electron transport chain and are self-sufficient (32, 33). The enzymes of the COX cascade are summarized in three excellent reviews, which may serve as introductions to this field (1, 2, 3).

In analogy, DOX of fungi oxidize LA to hydroperoxides, which can be isomerized by their fusion partners of three self-sufficient CYP subfamilies of linoleate diol synthases (LDSs), epoxy alcohol synthases (EASs), and allene oxide synthases (AOSs) (5, 34, 35). In plants, hydroperoxides produced by LOXs can also be isomerized by AOS, EAS, and many related enzymes of the Cyp74 family (36). Allene oxides of ALA can be further metabolized to jasmonates in both plants and fungi (4, 37, 38, 39).

Twenty-five years ago, Dr Smith and co-workers reported the tertiary structure of COX-1 with AA in the active site and described the lining amino acids of the substrate cavity in contact with the carbon chain, that is, the substrate recognition site (SRS) (40, 41, 42, 43). This was accomplished by replacement of heme iron with cobalt to halt the catalytic reaction. Reports on 3D structures of COX have since then been expanded in the Protein Data Bank (PDB), which today contains almost 40 tertiary structures of COX-1 and COX-2, including nonsteroidal anti-inflammatory drugs in the active site in many of them (44, 45, 46, 47, 48, 49, 50). This includes 3D structures of COXs of man, mouse, and sheep. COXs are also found in vertebrates, corals, and even in red algae (51, 52, 53). An ancient bacterial peroxidase may have evolved into the peroxidase-COX superfamily (54). The DOXs of fungi and cyanobacteria have added new information on this evolution (35, 55).

The fungal DOXs were first detected with the isolation of two characteristic metabolites, 8*R*-hydroxylinoleic acid and a related diol, 5*S*,8*R*-dihydroxylinoleic (5*S*,8*R*-DiHODE) acid in *Laetisaria arvalis* and *Aspergillus nidulans* (56, 57, 58). Studies of the take-all fungus of wheat (*Gaeumannomyces graminis*) revealed that LA was sequentially oxidized to 8*R*-hydroperoxylinoleic acid (8*R*-HPODE) and isomerized to a diol, 7*S*, 8*S*-DiHODE, by intramolecular hydroxylation (26). Purification and sequencing of this enzyme, 7,8-LDS, uncovered two heme cofactors per molecule and homology with COX-1 at the N-terminal half of the sequence (59, 60). Biosynthesis of 8*R*-HPODE was also associated with a tyrosyl radical and formation of a ferryl intermediate (compound I) in analogy with COX (27). This work and the discovery of MnLOX were summarized in a PhD thesis by C. Su (61). At this time, Dr Smith was the world-leading authority in the COX research field. Dr Smith accepted an invitation to visit Uppsala University and to act as the faculty opponent on October 1, 1999. Dr Smith’s attendance and scientific discourse made the dissertation an extraordinary event.

**Figure 1:**
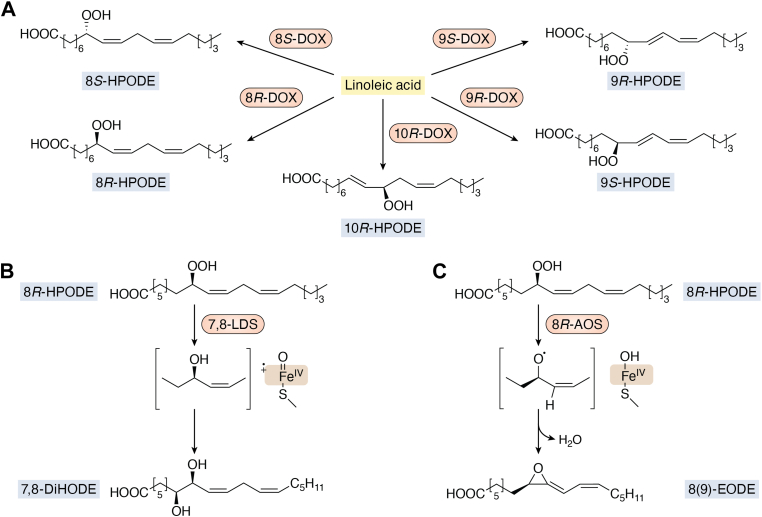
**Overview of the oxidation of LA by five fungal DOXsand the transformation of 8*R*-HPODE by 7,8-LDS and 8*R*-AOS.***A*, oxidation of LA by 10*R*-, 8*R*-, 8*S*-, 9*S*-, and 9*R*-DOX to five HPODE. *B*, intramolecular hydroxylation of 8*R*-HPODE by 7,8-LDS to 7*S*,8*S*-DiHODE by heterolytic cleavage of the hydroperoxide with oxidation of heme iron to compound I with a porphyrin cation radical as indicated. *C*, isomerization of 8*R*-HPODE to an allene oxide, 8*R* (9)epoxy-8,12*Z*-dienoic acid (8(9)-EODE), by homolytic cleavage of the hydroperoxide with oxidation of heme iron to compound II, hydrogen abstraction, and release of water. AOS, allene oxide synthase; COX, cyclooxygenase; DiHODE, dihydroxy-octadecadienoic acid; DOX, dioxygenase; HPODE, hydroperoxylinoleic acid; LA, linoleic acid; LDS, linoleate diol synthase.

The fungal LDS family has now been extended and combines 8*R*-, 8*S*-, 9*R*-, 9*S*-, and 10*R*-DOX with nine CYP fusion companions (62, 63, 64, 65, 66, 67, 68). The oxidation of LA by these five DOXs and the transformation of 8*R*-HPODE by two CYP fusion partners to a diol (7*S*,8*S*-DiHODE) and to an allene oxide (8*R* (9)epoxy-9,12*Z*-octadecadienoic acid) are summarized in Figure 1. 8*R*-HPODE can also be converted by intramolecular hydroxylation to 5,8-, 6,8-, and 8,11-DiHODE by the corresponding three LDSs and all four enzymes are designated 8*R*-LDS.

The LDS family is of scientific interest for three main reasons. First, these enzymes are produced by devasting fungal pathogens of wheat, rice, and other basal crops and participate in fungal reproduction and plant–fungal interactions (7, 10, 11, 15, 57, 69). Second, the DOXs are related to COXs and extend information on the catalytic properties of the peroxidase-COX superfamily. Third, their CYP partners illustrate the diversity of the LDS family with the transformation five different hydroperoxides of LA by nine subfamilies of CYP. An overview of the biological functions of LDS is outside of this minireview, but these enzymes participate in cellular differentiation, quorum-sensing, formation of appressoria, and other pathophysiological events (5, 10, 11, 14, 15, 16, 69, 70, 71, 72).

Unfortunately, we lack structural information and cryo-EM or X-ray crystallography for 3D analysis will likely be demanding. The recent improvements of computed structure models (CSMs), which include AlphaFold 2 (AF2) and related methods of artificial intelligence (AI) learning (73, 74, 75), may provide an alternative insight into the catalytic sites of LDS.

CSM can now predict the 3D structures of proteins with revolutionary precision (76, 77). AF2 was introduced in 2021 (73) and shared the Chemistry Nobel Prize for protein structure prediction and for protein design 3 years later. The AlphaFold Protein Structure Database contains over 220 million entries and a million have so far been included in the PDB (78). The last release, AF3, can also predict complex biomolecular interactions (79). All CSMs have nevertheless significant limitations. They do not provide any information on catalytic metals, prosthetic groups, posttranslational modifications, or the position of substrates (76).

AF2 models use a predicted local-distance difference test (pLDDT; 0–100) to describe confidence as a measure of likelihood (73). AF2 models with confidence in the upper range (average pLDDT > 90) are often considered to be of atomistic quality (73, 76). A recent comparison of 102 disparate pairs of high-quality X-ray crystal structures in two space groups with their AF2 models with very high confidence (pLDDT > 90) revealed median Cα atom errors of 0.3 and 0.6 Å, respectively. Large Cα atom errors (>2.0 Å) were twice as common in the AF2 models than in the pairs of proteins in different space groups, ∼10 *versus* ∼5% (77), which also illustrate the limited precision even of high-quality X-ray crystal structures. Furthermore, 93% of the predicted side chains were in agreement with experimental observations.

Amino acid residues of the LDSs were initially studied by site-directed mutagenesis of the DOXs based on multiple sequence alignment with COX and later by SWISS-MODEL with COX as a template or related methods ((62, 64, 67, 68, 80)t, (81)). These CSM were of limited accuracy in comparison with AF2 (73, 76). The CYP superfamily is structurally diverse and the CYP fusion partners lacked suitable CYP templates for model building. They were therefore mainly investigated by multiple sequence alignments. We therefore have limited knowledge of the SRSs and the environment of the heme-thiolate metal centers. An analysis based on recent improvements of CSMs will be necessary for re-interpretation of previous investigations and mt also generate novel hypotheses. This seems correct as “AF2 predictions often have very good stereochemical characteristics, making them excellent hypotheses for local structural features,” but they do not replace experimental structural determinations (77).

This minireview will compare the catalytic properties of fungal LDSs with five DOXs and nine CYP partners and a related cyanobacterial 10*S*-DOX with AF2 models of high confidence (pLDDT > 85). The enzymes were selected to illustrate a range of catalytic activities and their predicted 3D structures for a comparison with COX and CYP in the AA cascade. A larger number of fungal DOX-CYP fusion enzymes and their AF2 models were examined in greater details in two recent reports (34, 35). The most important unresolved questions are the position of fatty acids in the DOXs and the hydroperoxy fatty acids in the CYP fusion partners, and also the control of homolytic and heterolytic scission of oxygen–oxygen bonds. Focus of this review will therefore be on the oxygenation mechanisms, for example, the position of fatty acids for hydrogen abstraction, the orientation and position of O_2_ insertion, the subsequent intramolecular oxidations to diols or dehydration to allene oxides, and the heme-thiolate environment for the scission of oxygen–oxygen bonds. AF2 models may also provide structural information for a comparison of subfamilies of DOXs and their CYP fusion partners (8*R*-LDS, 8/9-AOS, and 10*R*-EAS) (34, 35). These models were examined with the PyMOL Molecular Graphics System (v. 3.1; *Schrödinger*, *L., & DeLano*, *W*. (*2020*); https://pymol.org/) and readers interested in this methodology will find technical support on the internet (*e.g.*, PyMOL Wiki, PyMOL Ross).

## 3D structures and AF2 models of COXs and DOXs

### 3D structures and AF2 models of COXs

A comparison of the resolution of the 3D structures of two ovCOX-1, two muCOX-2, and one huCOX-2 and the confidence (pLDDT) of their AF2 models is illustrated in Table 1. Superposition with the AF2 models (pLDDT 92.3–93.8) in PyMOL showed that the Cα atoms aligned with rmsd between 0.23 to 0.48 Å. The fact that the A and B chains in the X-ray crystals of ovCOX-1, huCOX-2, and muCOX-2 aligned with rmsd between 0.10 and 0.22 Å demonstrates the precision of the AF2 models. In conclusion, the difference between the Cα atom fold of 3D structures and their AF2 models appeared to be relatively small, but the AF2 models were not quite as perfect as these data may suggest.

**Table 1:** Comparison of the 3D structures of five of COX with their AF2 models

COX	PDB ID	Resolution (Å)	AF2 ID	pLDDT	Rmsd (Å)
ovCOX-1	2AYL	2.00	P05979	93.7	0.23
ovCOX-1	1IGZ	2.90			0.40
muCOX-2	4PH9	1.81	Q05769	92.3	0.38
muCOX-2	3HS5	2.10			0.48
huCOX-2	5F19	2.04	P35354	93.0	0.44

The rmsd were obtained by superposition of 3D structures and AF2 models. COX with PDB ID 2AYL was recorded in complex with flurbiprofen (45), 1IGZ with LA (42), 4PH9 with ibuprofen (47), 3HS5 with AA (85), and 5F19 with the aspirin-acetylated COX-2 (49). AF2 IDs have been abbreviated to UniProt ID (*e.g.*, AF-P05979-F1-model_v4 to P05979).

AA, arachidonic acid; AF2, AlphaFold 2; COX, cyclooxygenase; LA, linoleic acid; PDB, Protein Data Bank; pLDDT, predicted local-distance difference test.

The 3D structures of ovCOX-1 (2AYL) aligned in superposition with the 3D structures of huCOX-2, muCOX-2 (4PH9), and muCOX-2 (3HS5) with rmsd 0.61, 0.62, and 0.72 Å, respectively. The AF2 model ovCOX-1, however, aligned with the AF2 models of huCOX-2 and muCOX-2 with rmsd of only 0.30 and 0.31 Å, respectively. In addition, muCOX-2 (4PH9) and huCOX-2 (5F19) aligned with rmsd 0.40 Å, but their AF2 models were almost identical (rmsd 0.14 Å). AF2 models therefore likely interpreted 3D structures in the same way, which aligned them with lower rmsd than the experimental 3D structures (35). This error is presumably enhanced when the amino acid sequences are quite similar (87% sequence identity (seq. ID) between mu- and huCOX-2 and ca 60% between COX-1 and COX-2).

### Overview of AF2 models of DOXs

**Figure 2:**
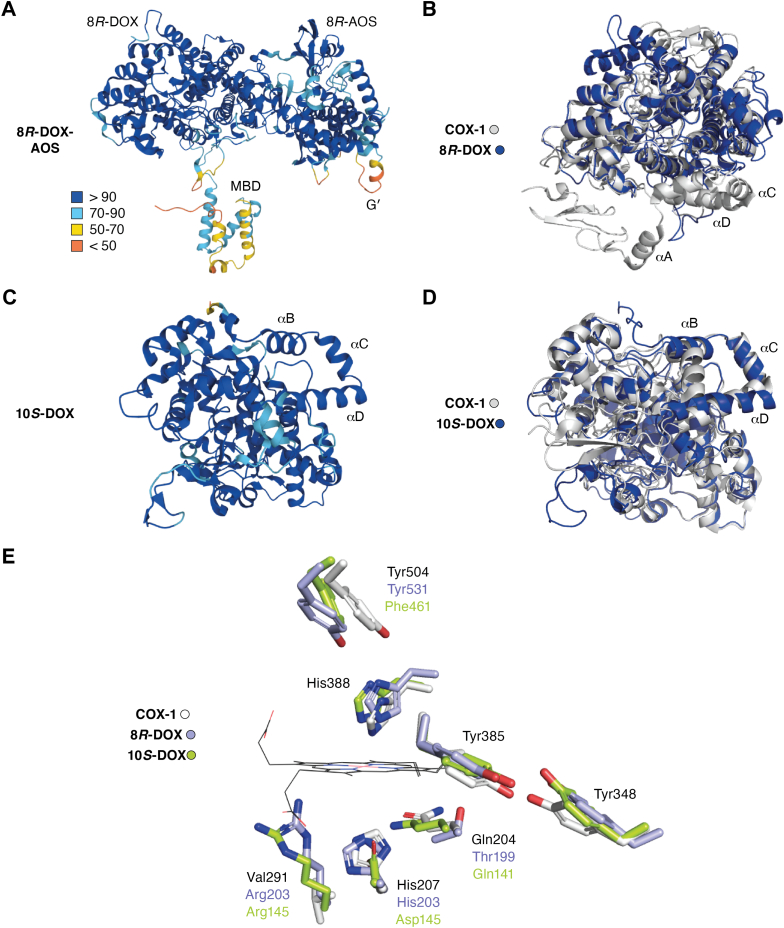
**AF2 models of 8*R*-DOX-AOS, 10*S*-DOX, and a comparison with COX-1.***A*, the AF2 model of 8*R*-DOX-AOS (pLDDT 90.2) with the color code of pLDDT values to the *left*. *B*, superposition of COX-1 in *gray* with the 8*R*-DOX as in (*A*) but without the MBD and the 8*R*-AOS sequences. Three of the four α-helices (αA-αD) of the MBD of COX-1 are marked. *C*, the AF2 model of the cyanobacterial 10*S*-DOX (pLDDT 94.5) with three α-helices of the MBD (marked αB-αD). *D*, superposition of COX-1 in *gray* with 10*S*-DOX in (*C*) showed alignment of αB-αD and many of the following α-helices. *E*, seven amino acids at key positions at the heme cofactor and at the catalytic center of COX-1 in superposition with the seven residues of 8*R*-DOX in *blue* and 10*S*-DOX in *green*. The residues of COX-1 are listed and also those of 8*R*- and 10*S*-DOX when the residues differ. AOS, allene oxide synthase; COX, cyclooxygenase; DOX, dioxygenase; MBD, membrane-binding domain; pLDDT, predicted local-distance difference test.

The AF2 model of 8*R*-DOX-AOS (average pLDDT 90.2) with the membrane-binding domain (MBD) and the two catalytic parts, 8*R*-DOX and 8*R*-AOS, are shown in Figure 2*A*. The catalytic domains were predicted with high/very high confidence except mainly for a loop with the α-helix G' (αG′) of the 8*R*-AOS (34, 35). The MBD was also predicted with variable confidence. Superposition of COX-1 with the 8*R*-DOX sequence revealed that the α-helical structures were largely conserved (Fig. 2*B*), but the MBD of COX-1 with the α-helices A-D (αA-αD) had no counterpart in 8*R*-DOX (*cf.* Fig. 2, *A* and *B*). COX-1 and COX-2 are also microsomal. Recombinant LDS are soluble, but the native prototype (7,8-LDS) was solubilized from acetone powder of mycelia and relatively loosely associated with membranes (59, 62).

The prokaryotic 10*S*-DOX contains only 542 amino acids and the fungal DOXs between 1050 and 1170 mainly due to variable lengths of the MBDs. 10*S*-DOX has about 50 less amino acids residues than COX and the AF2 model (pLDDT 94.5) was almost entirely predicted with high/very high confidence (Fig. 2*C*). The catalytic domain of COX contains 17 α-helices (labeled α1–α17) (50). Superposition with ovCOX-1 revealed a conserved α-helical structure from αB of the MBD to the last, α17 (Fig. 2*D*); the three α-helices of the MBD of 10*S*-DOX thus aligned with αB-αD of COX-1 (Fig. 2*D*).

The catalytic domain of COX show ∼25% seq. ID with 8*R*-DOX and ∼31% with the cyanobacterial 10*S*-DOX. They both align with COX with rmsd 1.2 Å (35). Many residues at key positions vary between COX and the eukaryotic and prokaryotic DOXs, but three are retained, namely, the proximal His388 ligand of the heme iron, the catalytic Tyr385, and the nearby central Tyr348 of COX (Fig. 2*E*). The distance between their phenylic oxygens of Tyr348 and Tyr385 varies between 2.4 and 2.7 Å in ovCOX-1 and muCOX-2, respectively, and they may form hydrogen bonds (82). These phenylic oxygens are further apart (∼7 Å) in the AF2 models of the fungal DOXs but they differ by only 3.1 Å in 10*S*-DOX (Fig. 2*E*).

The heme cofactor can be oxidized by peroxides to an intermediate (compound I), which can oxidize the nearby catalytic Tyr385 to a radical and also a distant Tyr504 (24). The latter is retained in 8*R*-DOX, but replaced by Phe in 10*S*-DOX (Fig. 2*E*). 8*R*-DOX also generates a tyrosyl radical and ferryl intermediates in analogy with COX (27). COX, 8*R*-DOX, or 10*S*-DOX differ at the position of the distal His207 heme iron ligand and at the nearby Val291 and Gln204 residues (Fig. 2*E*) (34, 55). The lining residues of the heme cavity of COX and the peroxidase activity have been investigated in detail (83). There is little information on the peroxidase activity of the DOXs; the unique and conserved Arg residue at the position of Val291 of COX-1 may merit investigation (Fig. 2*E*).

### AF2 models of LDSs

Two prototypes of the LDS family are 8*R*-DOX-7,8-LDS and 8*R*-DOX-5,8-LDS, which have been studied in detail (*vide infra*). They were mainly predicted with high/very high confidence of both the DOX and CYP domains. The N-terminal end with the MBD and a peripheral loop with αG′ of the CYP domains were predicted with less confidence, which reduce the average pLDDT (34, 35). The global pLDDT of all AF2 models of LDS were reduced in this way.

The α-helices of the eukaryotic DOXs aligned with catalytic domain of COX essentially as illustrated with 8*R*-DOX above (Fig. 2*B*). The α-helical bundles of 7,8-and 5,8-LDS aligned in superposition with α-helices of P450 BM3 (from αB to αL) with largely conserved fold of Cα atoms of the major α-helices (αD, αE, αI, αL, αK, and αL) (34). 8- and 9-AOS and 12*R*-EAS also aligned in this way. A selection of five different CYP fusion companions (seq. ID 21–45%) aligned in superposition with a deviation of Cα atom fold (rmsd) between 0.85 and 1.6 Å, but they aligned with the 3D structures of two prototypes, PGIS and P450 BM3, with rmsd >4 Å (34).

### α-Helical architectures of DOXs and COXs

The MBDs of LDSs (between 145 and 230 residues) were defined from the end of the N-terminal signal peptides (∼20 residues) to a conserved triad (ProAspPro) at the first α-helix of the catalytic domains, and the DOX and CYP borders at a conserved Pro residue between them (34, 35). 8*R*-DOX-5,8-LDS (B0Y6R2) thus consisted of 8*R*-DOX (D168-R646) and 5,8-LDS (P647-E1079) with 478 and 432 amino acids, respectively.

8*R*-DOX aligned in superposition with α-helices of ovCOX-1 from α2 to the last one, α17. COX-1 contained three α-helices (α7, α8, and α9) without apparent homologs in 8*R*-DOX and the latter also contained three unique α-helices (34). The third (P628-L638) was positioned after α17 and required for expression (84). The α-helices of 8*R*-DOX were largely retained in 8*S*-, 9*R*/9*S*-, and 10*R*-DOX (35).

The catalytic domains of ovCOX-1 and muCOX-2 aligned with rmsd 0.49 Å and with the catalytic domains of 9*S*-, 9*R*-, and 8*R*-DOX with rmsd of 1.2 to 1.3 Å (35). Six members of the 8*R*-DOX-LDS subfamily (seq. ID 49–78%) aligned with rmsd between 0.41 and 0.78 Å, but with larger deviations (0.7–1.6 Å) between subfamilies of 8-, 9-, and 10*R*-DOX with seq. ID 35 to 54% (35).

### SRS of 9- and 8-DOX

**Figure 3:**
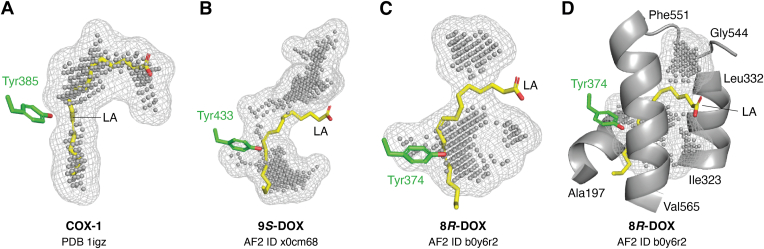
**A comparison of the substrate cavity of the ovCOX-1:LA complex with the AF2 models of 9*S*-DOX, 8*R*-DOX, and three surrounding α-helices of the substrate cavity of 8*R*-DOX.***A*, the curved substrate cavity of ovCOX-1 with the catalytic Tyr385 in *green* close to C11 of LA. The nonbonding spheres (radius 0.25 Å) in *gray color* indicate the volume of the cavity and covered LA in *yellow* except at C11 and part of the carboxylate group. *B*, the AF2 model of the substrate cavity of 9*S*-DOX with the catalytic Tyr433 in *green* close to C11 of LA in superposition with the COX-1:LA complex (PDB: 1IGZ). *C*, the substrate cavity of 8*R*-DOX with the catalytic Tyr374 close to C11 of LA of the COX-1:LA complex. *D*, the substrate cavity of 8*R*-DOX with Tyr374 was lined by residues of three α-helices, which were labeled the *bottom* (A197-L202), *central* (I323-L332), and *terminal* (loop G544-F551, α-helix F551-V565) motifs of the SRS and the latter two are summarized in Table 2. Cavities were analyzed with the PyMOL plugin CavitOmiX (radius factor 0.68 (DOX) and 0.83 (COX)) as described (35). AF2 IDs were abbreviated to UniProt ID (in *lowercase letters* for clarity). AF2, AlphaFold 2; COX, cyclooxygenase; DOX, dioxygenase; LA, linoleic acid; SRS, substrate recognition site.

LA was positioned in carboxylate-out orientation in the curved (“L-shaped”) substrate cavity of ovCOX-1 as illustrated by the COX-1:LA complex (PDB: 1IGZ; (42)) (Fig. 3*A*). LA was almost completely covered by nonboding spheres (radius 0.25 Å), which indicate the volume of the cavity, and hardly visible except at C11 close to the catalytic Tyr385 and at the carboxylate group at the entrance. The substrate cavity of 9*S*-DOX was less curved (Fig. 3*B*). This cavity covered LA in superposition except for protruding carbons, C1-C3 and C17-C18 (35). The catalytic Tyr433 was positioned close to C11 of the COX-1:LA complex in superposition. The entrance of the 9*S*-DOX cavity was not visualized at the surface and appeared to be blocked by α-helical residues of the Leu83-Ala99 sequence, which were predicted with high confidence (35). They aligned partly with αB of COX-1. The contour of the substrate cavity of 9*R*-DOX was predicted with a distinct entrance at the protein surface and with Tyr431 in the very same position as Tyr433 of 9*S*-DOX (35).

The substrate cavity of 8*R*-DOX consisted of two chambers, which were connected by a relatively narrow passage (Fig. 3*C*). This cavity covered C4-C16 of LA in superposition and with the catalytic Tyr374 close to C11 of LA. As discussed below, Tyr374 will abstract hydrogen from C8 and this carbon appears to be accessible with LA in the opposite orientation. The position of entrance to the substrate cavity differed from that of COX as illustrated by superposition of the COX:LA complex. The contour of the substrate cavities of 8*R*-DOX differed only slightly from 10*R*-DOX (35).

The substrate cavity of 8*R*-DOX was lined by three α-helices, which provided residues to the SRS (Fig. 3*D*). The α-helix at the bottom (A197-L202) contained the distal His ligand of heme iron (*cf.* Figs. 2*E* and 3*D*). The conserved Ala and Ile residues of the SRS of this α-helix (AxxIthd of DOX) were largely retained in fungal DOX, but replaced by F^205^thqF of COX and F^143^tdsF of 10*S*-DOX. The next α-helix (I323-L332) of 8*R*-DOX contained the central Tyr residue (Tyr348 of COX; *cf.* Figs. 2*E* and 3*D*). This α-helix and an N-terminal loop connected to an α-helix (G544-F551-V565) also contributed with many catalytically important residues. These SRS were labeled the central and terminal motifs, and they were predicted with high/very high confidence (35). These SRSs of two COXs and seven DOXs are summarized in Table 2 along with conserved residues close to the catalytic Tyr and a conserved Lys residue of the eukaryotic DOXs.

**Table 2:** SRSs of the COX:LA and COX:AA complexes and the AF2 models of SRS of six fungal and one bacterial DOX

Enzyme	Central motif	Tyr seq.	Lys	Terminal motif
COX				
ovCOX-1:LA	VIxxY^348^V^349^xxLS^353^xYxxxL	L^384^Y^385^xW	-	F^518^xxxMIxxG^526^AxFS^530^LxG^533^L^534^
muCOX-2:AA	VxxxY^348^V^349^xxLS^353^GYxxxL	L^384^Y^385^xW	-	R^513^ AIF^518^xxxMVExG^526^AxFS^530^LxG^533^L^534^
Fungal DOX				
9*R*-DOX	YVxxSLxxY^394^LxxL^398^T	Y^441^xF	K^607^	GCGG^616^CPxxVGR^623^xVF^626^xxAVxLV
9*S*-DOX	YIxxCLxxY^386^LxxI^390^T	Y^433^xF	K^600^	GSGI^609^MPxYxVGR^617^xVL^620^xDAVxLV
8*R*-DOX	YIxxV^325^LxxY^329^VxxIL^334^	Y^376^xW	K^540^	GSGL^549^CP^551^xxxxS^556^xxI^559^LxDAVxLV
8*S*-DOX	I^306^LxxY^310^VxxIL^315^	Y^357^xW	K^521^	GSGLCA^532^xxxMA^537^xxI^540^LxDAVxLV
10*R*-DOX-1	IxxTLxxY^383^LxxIV^388^	Y^427^xW	K^590^	GVGI^599^AP^601^xxxISxxV^609^LxDAVxLV
10*R*-DOX-2	IxxTLxxY^357^VxxII^362^	Y^405^xW	K^568^	GVGIAP^579^xxx ISxxV^587^LxDAVxLV
Bacterial 10*S*-DOX				
10*S*-DOX	IMxxY^305^IxxIT^310^PyxxxL	FxxxY^342^	-	G^469^RxxSxIPAxxARxI^483^GxDA^487^FxQ^490^A

ovCOX-1 was crystallized with LA (PDB: 1IGZ) and muCOX-2 with AA (3HS5_B) (42, 85). The UniProt ID numbers of the DOX are from top to bottom: Q0CW98 (pDDT 87.9), X0CM68 (85.6), Q9UUS2 (86.4), F9XMQ7 (89.6), Q4WY82 (90.1), W9JMD9 (88.1), and B2J5P4 (94.5). The lining residues of the substrate cavities were determined by a PyMOL plugin CavitOmiX with radius factor 0.68 to 0.70 (DOX) and 0.83 (COX) as described (35). The numbered residues indicate conserved positions or catalytic relevance.

AA, arachidonic acid; AF2, AlphaFold 2; COX, cyclooxygenase; DOX, dioxygenase; LA, linoleic acid; SRS, substrate recognition site.

To sum up, AF2 models predicted the catalytic Tyr residues of the eukaryotic 8-, 9-, and 10-DOX closely aligned with the phenylic oxygen close to C11 of the COX-1:LA complex in superposition. The central motif was largely retained in the form of a variation of nonpolar residues at fixed positions relative to the central Tyr residue (Table 2). Many of them have been analyzed by replacements (*vide infra*). The terminal motif was variable with only few conserved residues but the Cα atoms of the α-helical part of this motif of COX and the DOXs nevertheless partly aligned. Opposite orientations of LA in the active sites of 8- and 10*R*-DOX *versus* 9-DOX might explained hydrogen abstraction at two positions (C8 and C11, respectively) rather than the differences in the SRSs (*vide infra*).

### SRS of COX

COXs are heterodimers with a catalytic and an allosteric subunit with identical 3D structures, but they bind AA in opposite orientations (23, 85). Both units can form the tyrosyl radical, but the catalytic unite can only oxidize AA (86). The substrate cavity of COX-2 is wider than COX-1 in the upper region near the entrance (48, 49). The central and terminal motifs and few other positions of the SRSsof ovCOX-1 with LA and muCOX-2 with AA in the active site are illustrated in Table 2. Most of these lining residues have been investigated by replacements (2, 3, 23, 40, 41, 43, 87, 88).

Following many modifications of the active site, COX will usually oxidize AA to PGH_2_ but also to the two side products, 11*R*- and 15*S*-hydroperoxyeicosatetraenoic (HPETE) acids (2, 3, 40, 41). At least four residues (V349, W387, G533, and L531) positioned AA for hydrogen abstraction by the catalytic Tyr385 as replacements reduced or abolished catalytic activities (2, 40). Replacements of some of them and also Leu384, Phe518, Gly526, Ser530, and Leu534 could increase the formation of the side products and also induce biosynthesis of 15*R*-HPETE and two metabolites with 8,9-11,12-diepoxy-13*R*/15*R*-hydroperoxy structures (40, 41, 87, 88). Arg120 at the entrance of the catalytic subunit can stabilize the position of AA by tethering of the carboxylate group (3, 43). The target for acetylation of COX by aspirin is Ser530 (3, 89), but this Ser residue is not conserved in the DOX (35). Residues for site-directed mutagenesis of 8*R*-DOX were initially selected by sequence alignment with catalytic important amino acids of COX, for example, His207, Tyr348, Tyr385, Trp387, and Tyr504 (*cf.* Fig. 2*E*) (90).

## 9*R*- and 9*S*-DOX and the AF2 models

### Oxidation of fatty acids by 9-DOX and ovCOX-1

**Figure 4:**
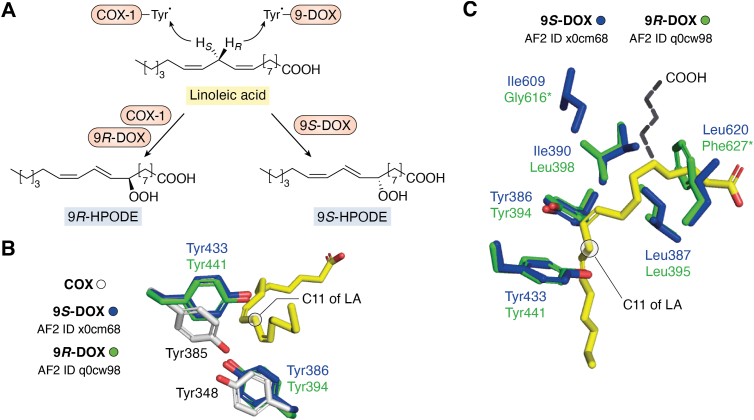
**Overview of the oxidation of LA by COX-1, 9*R*- and 9*S*-DOX and active site residues.***A*, ovCOX-1 and 9*R*/9*S*-DOX oxidized LA at C9 after abstraction of the 11*S* and 11*R* hydrogens by the tyrosyl radicals, respectively. *B*, Tyr348, Tyr385, and LA (*yellow*) of the active site with LA of the ovCOX-1: LA complex with residues in *white* with superposition of the corresponding residues of 9*S*-DOX in *blue* (Y386, Y433) and 9*R*-DOX in *green* (Y394, Y441) in relation to C11 of LA. *C*, superposition of the catalytic and central Tyr residues and four hydrophobic residues of 9*S*-DOX (in *blue*; numbered in *bold*) and 9*R*-DOX (in *green*) and LA of the ovCOX-1:LA complex (in *yellow*) with the catalytic Tyr residues near C11 of LA, but the α-side chain of LA must adopt another configuration in nine-DOX than in COX-1 due to stereochemical constraints (F627 and L620). Replacements of residues marked ∗ altered O_2_ insertion at C8 and C10. AF2 IDs were abbreviated to UniProt ID (in *lowercase* letters for clarity). AF2, AlphaFold 2; COX, cyclooxygenase; DOX, dioxygenase; LA, linoleic acid.

9*S*-DOX and ovCOX-1 oxidized LA by abstraction of the 11*R* and 11*S* hydrogens of LA, respectively, followed by antarafacial oxygenation of C9 to produce 9*S*-HPODE and 9*R*-HPODE, respectively (Fig. 4*A*) (64, 65, 67, 91); ovCOX-1 formed 9*S*-HODE (9%) and 13*S*-HODE (17%) (92). 9*R*-DOX also abstracted the 11*R* hydrogen, but catalyzed suprafacial hydrogen abstraction and O_2_ insertion in analogy with 8*S*-DOX (*vide infra*).

The phenyl oxygens of the catalytic Tyr residues of 9*R*- and 9*S*-DOX aligned, but they were separated from Tyr385 of the COX-1:LA complex by ∼4 Å (Fig. 4*B*). This separation might support abstraction of the 11*R* and 11*S* hydrogens of LA by 9-DOX and COX-1, respectively, but rotation of LA along the carbon chain and other steric factors may optimize the reaction.

9*S*-DOX-AOS (X0CM68) oxidized LA, ALA, 18:3*n*-6, and the Gly, Ile, and Trp conjugates of LA at C9. 20:2*n*-6 was oxidized at C11 (9 + 2) by 9*S*- and 9*R*-DOX. 9*R*-DOX of *Fusarim oxysporum* (F9FU71) also oxidized the Trp and Ile conjugates of LA at C9 (93, 94). 9*S*- and 9*R*-DOX therefore likely position fatty acids in carboxylate-out configuration analogy with COX.

### Overview of α-helical fold and SRS of 9-DOX

The protein Cα atom fold of 9*R*- and 9*S*-DOX was highly conserved (rmsd 0.34 Å; seq. ID 53%). 9- and 8*R*-DOX also aligned closely (rmsd 0.58–0.60 Å; seq. ID ∼37%). Important motifs of the SRS of 9-DOX are summarized in Table 2.

9*S*- and 9*R*-DOX were predicted with Arg617 and Arg624, respectively, at the entrances to the substrate cavities (Table 2). They might tether the carboxyl group of LA in carboxylate-out position in analogy with AA and Arg120 of COX-1 (3, 23). The characteristic YxF sequences with the catalytic Tyr of 9-DOX (Table 2) correspond to the YxW sequences of COX, 8-, and 10*R*-DOX. The F435W replacement of 9*S*-DOX did not alter the oxygenation to 9*S*-HPODE (67). For comparison, the W378F replacement abolished the activity of 8*R*-DOX, but the W387F and W387Y replacements of COX-2 were less dramatic with an increased formation of the side products (88, 90).

The different oxygenation of C9 by 9*R*- and 9*S*-DOX might be related to pairs of hydrophobic residues, for example, L398/I390, G616/I609, and F627/L620 (Table 2; Fig. 4*C*). The last pair was situated so that the α-side chain of LA must adopt another configuration than in the COX-1:LA complex. The F627L replacement of 9*R*-DOX led to biosynthesis of 9*S*-HPODE as the main products, and the double mutant of 9*R*-DOX (G616I/F627L) increased this further to >90% 9*S*-HPODE (95).

## 8*R*-, 8*S*-, and 10*R*-DOX and the AF2 models

### Oxygenation of fatty acids by 8-DOX

**Figure 5:**
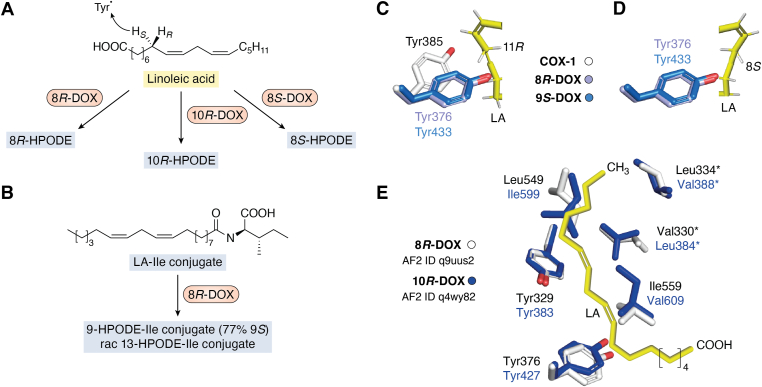
**The mechanism of oxidation of LA by eight-DOX and 10*R*-DOX and a comparison with nine-DOX.***A*, oxidation of LA by 8- and 10*R*-DOX occurred by hydrogen abstraction at C8 followed by insertion of O_2_ at C8 or C10 of LA, respectively. *B*, oxidation of the LA-Ile conjugate by 8*R*-DOX (Q0CPQ2) produced 9-HPODE-Ile (77% 9*S*) and rac 13-HPODE-Ile in a ratio of 2:1. *C*, Tyr385 of COX-1, Tyr376 (8*R*-DOX), and Tyr433 (9*S*-DOX) and a partial sequence of LA of the COX:1-LA complex with 9*S*-DOX (Tyr433) in position to abstract the 11*R* hydrogen. *D*, Tyr residues of 8*R*- and 9*S*-DOX as in (*C*) but with LA in carboxylate-in position at the mirror image of LA in (*C*) and with 8*R*-DOX (Tyr376) in position to abstract the 8*S* hydrogen. *E*, a comparison of six residues of the active sites of 8*R*- and 10*R*-DOX, which may allow access of O_2_ to C8 or C10. 8*R*-DOX in *white* with *bold letters* and 10*R*-DOX in *violet*. Replacements of two pairs (marked ∗) altered the insertion of O_2_ between C8 and C10 (see text). LA might bind 8*R*- and 10*R*-DOX as indicated by the drawing. AF2 IDs were abbreviated to UniProt ID (in *lowercase letters* for clarity). AF2, AlphaFold 2; AOS, allene oxide synthase; COX, cyclooxygenase; DOX, dioxygenase; HODE, hydroxy-octadecadienoic acid; HPODE, hydroperoxylinoleic acid; LA, linoleic acid; LDS, linoleate diol synthase.

8*R*- and 8*S*-DOX abstracted the 8*S* hydrogen at C8 of LA but inserted O_2_ in different steric positions (Fig. 5*A*). The catalytic Tyr376 of 8*R*-DOX will therefore be close to C8 for hydrogen abstraction. 8*R*- and 8*S*-DOX showed a distinct substrate specificity for OA, LA, and ALA, but 18:3*n*-6 was not oxidized and unsaturated C_20_ fatty acids were poor substrates (25, 68).

8*R*-DOX-7,8-LDS and 8*R*-DOX-AOS oxidized the LA-Gly conjugate mainly at C8 but also at C9 (94). The Ile and Trp conjugates of LA were oxidized by hydrogen abstraction at C11 and insertion of O_2_ at C9 and C13 (93, 94, 96). 8*R*-DOX-5,8-LDS (Q0CQP2) formed the Ile conjugates of 9-HPODE (77% 9*S*) and rac 13-HPODE in a ratio 2:1 (Fig. 5*B*), but other 8*R*-DOX formed the 9*S*-HPODE-Ile conjugate with less stereospecific (62%) (93, 94). In conclusion, 8*R*-DOX will likely oxidize LA in carboxylate-in position in contrast to 9-DOX.

This conclusion was also supported the abstraction of the 11*R* and 8*S* hydrogens by 9- and 8-DOX, respectively (Figs. 4*A* and 5*A*), but this may not be obvious from the *R* and *S* labels. The positions of Tyr385 (COX-1), Tyr433 (9*S*-DOX), and Tyr376 (8*R*-DOX) are shown in relation to the 11*R* hydrogen of the COX-1:LA complex (Fig. 5*C*) and to the 8*S* hydrogen of LA in the opposite orientation at the position of the mirror image of LA (Fig. 5*D*). 9*S*-DOX is thus aligned for abstraction at the 11*R* hydrogen and COX-1 for the 11*S* hydrogen of LA in the COX-1:LA complex, and 8*R*-DOX (Tyr376) is in position for abstraction of the 8*S* hydrogen of LA in the opposite orientation (and the 11*R* hydrogen of the LA-Ile conjugate).

### SRS of 8*R*-DOX and COX

Part of the SRS of 8*R*-DOX is shown in Table 2. Residues with homology to key positions of COX were studied initially. The replacements of the distal heme ligand (H203Q) and the central Tyr residue (Y329F) only reduced catalysis, but it was abolished by Tyr329Leu and also by Tyr376Phe, Trp378Phe, and Tyr531Phe (Fig. 2*E*). Replacements of the conserved Lys540 (with L, Q, and R) at a solitary position of the SRS (Table 2) also abolished catalysis (90).

### Kinetic deuterium isotope effects at allylic positions

The C-D bond is stronger than the C-H bond, and classical theoretical calculations predict ^H^k_cat_/^D^k_cat_ ratios < 7, but much larger ratios (>20) can be explained in quantum theory by hydrogen tunneling through the energy barrier (97). COX-1, 8*R*-DOX, and 9*S*-DOX oxidized [11*S*-^2^H]LA, [8*S*-^2^H]LA, and [11,11-^2^H_2_]LA, respectively, by hydrogen and deuterium abstraction at bis-allylic positions with kinetic deuterium isotope effects of less than 5 (26, 64, 65, 67, 96, 98). The energy barrier for hydrogen abstraction at allylic positions is much higher than at bis-allylic carbons. This is illustrated by the low rate of oxidation of OA and 20:1*n*-6 by DOX and COX in comparison with LA and 20:2*n*-6 and by the oxidation of [8,8-^2^H_2_]OA and [13,13-^2^H_2_] 20:1*n*-6 by 8*R*-DOX and COX-1, respectively, with large kinetic deuterium isotope effects (>20) (96). These demanding reactions might therefore be associated with hydrogen tunneling in analogy with the oxidation of LA by Fe- and MnLOX (96, 97, 99).

### Oxygenation of fatty acids by 10*R*-DOX

10*R*-DOXs are either fused to EAS as in 10*R*-DOX-2-EAS in plant pathogens or to a protein without the heme-thiolate ligand as in 10*R*-DOX-1 of Aspergilli (Q4WY82) (62, 66, 100). Both enzymes oxidized LA to 10*R*-HPODE as described in Figure 5*A*, but 10*R*-DOX-1 oxidized OA at C10 and 10*R*-DOX-2 mainly at C8. 10*R*-DOX-2 also oxidized 20:2*n*-6 and 20:3n-3 at C10 and C12 (62, 100). 10*R*- and 8*R*-DOX display similar the substrate specificities and 18:3*n*-6 was not oxidized (25, 68, 100).

### SRS of 8- and 10-DOX and oxygen insertion

The SRSs of 8*R*- and 8*S*-DOX were largely conserved at the central and terminal motifs (Table 2). Pairs of amino acid residues differ at three positions, I306/V325, A532/P551, and A537/S556, which might tentatively direct O_2_ insertion at C8 in the two steric positions (35), but experimental data are lacking. The central Tyr residues were followed by Leu384 in 10*R*-DOX-1, Val358 and Val407 in the two 10*R*-DOX-2, and by Val330 of 8*R*-DOX (Table 2). It would therefore be interesting to know whether Leu384 can shield C8 of OA for oxygenation by 10*R*-DOX-1.

The SRS of 10*R*-DOX-1 differed from 8*R*-DOX (Q9UUS2) in relatively few positions (Table 2). Four of them are illustrated in the active sites of 8*R*- and 10*R*-DOX-1 with a schematic view of LA in carboxylate-in orientation with C8 close to the catalytic Tyr residues (Fig. 5*E*). Replacements of Val with Leu, or *vice versa*, at the L384/V330 and V388/L334 positions (Q4WY82/Q9UUS2) increased and reduced the relative biosynthesis of 8*R*- and 10*R*-HPODE as expected from the sequence differences (100, 101). The V330L replacement of 8*R*-DOX increased the oxygenation at C10 threefold, and the L384V and V388L replacements of 10*R*-DOX the oxygenation at C8. These two positions can thus control O_2_ insertion at C8 and C10 by 8*R*- and 10*R*-DOX, respectively. The two homologous positions of COX-1, Val311 and Ser353, are also critical for position and stereospecificity (Table 2) (40, 43, 87, 89, 102). In the terminal motif, Ile483 of 10*S*-DOX corresponds to Val residues of 10*R*-DOX and to G526 of COX (Table 2). Whether the Ile and Val residues of 10*S*- and 10*R*-DOX support the stereospecific insertion of O_2_ merits investigation.

## 10*S*-DOX and the AF2 model

The 10*S*-DOX of the cyanobacterium *Nostoc punctiforme* produces 10*S*-HPODE, which can be transformed by a catalase-related and specific hydroperoxide lyase of a separate gene (55).

### 10*S*-DOX

10*S*-DOX oxidized OA and LA to 10*S*-hydroperoxides, but 18:3*n*-6 was not a substrate and 20:2*n*-6 was oxidized at a reduced rate (55). 8*R*- and 10*R*-DOX likely bind LA in carboxylate-in (head-first) orientation as discussed above and this seems to be a reasonable hypothesis also for 10*S*-DOX based on these catalytic properties. It is unknown whether 10*S*-DOX also abstracts the 8*S* hydrogen of LA in analogy with 8- and 10*R*-DOX and then catalyzes suprafacial hydrogen abstraction and oxygenation in analogy with 8*S*- and 9*R*-DOX. The AF2 model does not provide any information on this reaction as the catalytic Tyr342 of 10*S*-DOX was positioned near C11 of the COX-1:LA complex with the phenylic oxygen between these oxygens of Tyr385 (COX) and Tyr376 (8*R*-DOX) (*cf.* Fig. 2*E*).

### α-Helices of 10*S*-DOX, COX, and the SRS

The Cα atom fold of 10*S*-DOX and COX-1 aligned with rmsd of ∼1.2 Å (*cf.* Fig. 2*D*).

10*S*-DOX contains three homologs α-helices (αB-αD; Fig. 2, *C* and *D*) of the MBD of COX. The next α-helix (W84-N88) after αD of 10*S*-DOX aligned in superposition with α1 of ovCOX-1 (S138-N144) and the following α-helices then aligned with COX including the C-terminal α17, but 10*S*-DOX contained three additional α-helices (between α3 and α5) (35).

10*S*-DOX differed from both COX and eukaryotic DOX in terminal motif of the SRS. Only three residues (G469; D486A) aligned with the corresponding residues of 10*R*-DOX and neither of them with COX (Table 2). Gly526 and Ser530 of COX correspond to Ile483 and Ala487 of 10*S*-DOX, but the two Phe residues of the α-helix at the bottom of the substrate cavities, F^143^tdsF of 10*S*-DOX and F^205^thqF of COX, were conserved as discussed above.

### A comparison of 10*S*- and 10*R*-DOX

10*S*-DOX and 10*R*-DOX-1 aligned with 21% sequence identity and the Cα atom fold with rmsd 1.5 Å. Two replacements (L384V and V388L) of 10*R*-DOX-1 increased the oxygenation at C8 (Fig. 5*E*) (101). Ile306 and Thr310 at these positions of 10*S*-DOX might therefore be worthy of investigation (Table 2).

## Overview of LDSs and AOSs

The self-sufficient catalytic mechanisms of two prototypes, 7,8-LDS and 8*R*-AOS, are summarized in Figure 1, *B* and *C*. A key question is how the heterolytic and homolytic cleavage of the 8*R*-hydroperoxide group is controlled. Residues on both sides of the cysteine-bound heme iron might facilitate these reactions.

### The proximal face of the heme-thiolate cavity

The proximal face (“Cys pocket”) of the heme cavity is lined by six often conserved amino acid residues of man and fungi (FGxGx**R**x**C**xG) and bacteria (FGxGx**H**x**C**xG) on a loop up to the heme-thiolate ligand and followed by residues of αL (103). The two different residues (Arg and His) coordinate heme binding by hydrogen bonds (104). All LDS retained the HxC sequence of bacteria in, for example, HEC of 9-AOS and H(Q/A)C of 8-AOS (34). A majority of the DOX fusion partners also retained 2 to 3 additional residues of the six (34). Amide protons in the Cys pocket may neutralize the negative charge of the sulfur atom and stabilize the thiolate-iron coordination (105), but whether this can occur in 8*R*-LDS or 8/9-AOS is unknown.

### The SRS of the distal face of the heme-thiolate cavity

The SRS of CYP differ due to both sequence and spatial hypervariability (104, 106). The six original SRS motifs, which were based on the α-helical structures and β-sheets of CYP2 and also on bacterial prototypes, were subsequently extended to a large number of isoforms (107). The SRS of the CYP of the LDS family included homologs of the SRS1, SRS2, SRS4, and SRS5 of P450 BM3. The nonpolar residues of SRS2 at αF may form hydrophobic (π) interactions with the double bonds of HPODE, and polar and nonpolar residues of SRS4 at αI appear to be of particular importance for the position the hydroperoxide group at the heme iron for scission of the oxygen–oxygen bond (34).

### Tertiary structures of 5,8-LDS, 8*R*- and 9*R*-AOS

The α-helices of 5,8-LDS and 8*R*-AOS folded in superposition with rmsd ∼1.2 Å, but 9*S*-AOS aligned with rmsd 1.6 and 1.3 Å, respectively. 5,8-LDS and 8*R*-AOS thus appeared to be closely related with 9*S*-AOS as possibly a more distant homolog (34). The general Cα fold of these CYP fusion partners was retained as discussed above. Posttranslational modifications in fungi may modify 8*R*-LDS activities (90), but these enzymes can nevertheless be successfully expressed in *Escherichia coli*.

## 8*R*-LDS, 10*R*-EAS, and the AF2 models

### 8*R*-LDS

Seq. ID divided the four 8*R*-LDSs into three groups: 7,8-LDS of plant pathogens, 5,8-LDS of aspergilli, and 6,8- and 8,11-LDS of penicillium (34). The predicted SRS are summarized in Table 3. 5,8-LDS is illustrative of this subfamily. This enzyme catalyzed intramolecular hydroxylation of the 8*R*-hydroperoxides of 16:1*n*-7, OA, LA, and ALA, but not the 9*R*-hydroperoxide of 19:3*n*-3 (62). The different 8*R*-LDSs catalyze intramolecular hydroxylation of 8*R*-HPODE at C5, C6, C7, or C11 and with position and stereospecificity with two exceptions, 7,8-LDS (E3QVI1) and 8,11-LDS (B6HQI5) (108, 109).

**Table 3:** AF2 models of the SRSs of 5,8-, 6,8-, and 7,8-LDS, 10*R*-EAS, 8*R*/8*S*-AOS, and 9*R*/9*S*-AOS

Enzyme	Lining amino acids of heme-thiolate cavities					
	SRS1 αB′	SRS1a Loop αB′-αC	SRS-αE αE	SRS2 αF	SRS4 αI	SRS5 β1-2
5,8-LDS	W^676^	F^695^ML	-	F^795^xxxFY	PTxxGMxxN^887^QxQ	SVA^941^LxR
6,8-LDS	W^675^	F^694^ML	-	F^794^xxIFY	SxxSMxxN^886^QxQ	SVA^940^LPR
7,8-LDS	W^684^	F^703^CL	-	F^804^xxVFL	PVxxANVxN^897^QxQIxxM	AVA^950^VYR
10*R*-EAS	W^706^	F^721^ML	-	F^821^xxxFF	TxxAMxxN^914^QxQVxxQ	TFG^969^LYR
8*R*-AOS	W^657^	YM^670^L	FxxxFF^743^	F^770^xxxFL	TxxASxxT^861^QxQ	AxG^916^V
8*S*-AOS	V^657^xA	WM^672^L	HxxVTxxIF^745^	F^769^QxTFL	TxxAAxxI^863^QxQG	PAAxG^918^ALR
9*R*-AOS	V^743^xW	M^762^LxGD	MxxxF^836^	R^863^xxxFN	TxIGGVGT^957^PxG^960^V	SMEC^1012^TVR
9*S*-AOS	W^738^	M^756^L	NxxLLxxLF^830^	R^857^ xxxVN	LTAFGGxxV^958^PxT^961^A	SxQR^1009^NVR

Four SRSs were positioned at α-helices (B′, E, F, and I), SRS1a at a loop between αB′ and αC, and SRS5 at β1-2 (34). Residues were numbered to indicate conserved positions. Nonaligning residues were marked x. The heme-thiolate cavities were analyzed by a PyMOL plugin (CavitOmiX; radius factor 0.9), and listed residues are within 14 to 15 Å from the Cys/S of the heme-iron ligand (34). The UniProt ID numbers are from top to bottom: B0Y6R2 (pLDDT 89.6), S7ZN32 (89.4), E3QVI1 (86.1), W9JMD9 (88.1), J3K2W8 (90.2), F9XMQ7 (89.6), Q0CW98 (85.6), and X0CM68 (87.9).

AA, arachidonic acid; AF2, AlphaFold 2; AOS, allene oxide synthase; COX, cyclooxygenase; DOX, dioxygenase; EAS, epoxy alcohol synthase; LA, linoleic acid; LDS, linoleate diol synthase; pLDDT, predicted local-distance difference test; SRS, substrate recognition site.

### SRS of 8*R*-LDS

**Figure 6:**
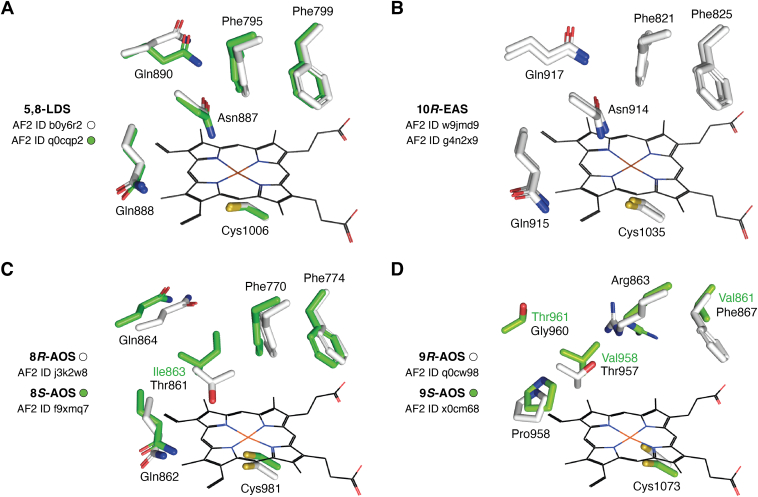
**AF2 models of the active sites of 5,8-LDS, 10*R*-EAS, 8*R*/8*S*-AOS, and 9*R*/9*S*-AOS with five residues of SRS2 and SRS4 and the heme-thiolate ligands.***A*, models of 5,8-LDS with residues in *white* (B0Y6R2; numbered) and in *green* (Q0CQP2). *B*, models of 10*R*-EAS (W9JMD9, numbered; G4N2X9) with all residues marked in *white*. *C*, models of 8*R*-AOS (in *white*; numbers in *bold*) and 8*S*-AOS (in *green*). The conserved Asn residues in (*A*) and (*B*) were replaced by Thr861and Ile863 of 8*R*- and 8*S*-AOS, respectively. *D*, models of 9*R*-AOS (in *white*, numbers in *bold*) and 9*S*-AOS (in *green*, unique residues numbered). The conserved Asn residues in (*A*) and (*B*) were replaced Thr957 and Val958 in 9*R*- and 9*S*-AOS. 9-AOS also differed from 5,8-LDS at three other positions (F867/V861; T957/V958; G960 (not visible)/T961). Heme of P450 BM3 (2HPD(104)) was shown in (*A–D*) in superposition. The heme-thiolate cavities were investigated by a PyMOL plugin (CavitOmiX; radius factor 0.83) as described (34). AF2 IDs were abbreviated to UniProt ID (in *lowercase letters* for clarity). AF2, AlphaFold 2; AOS, allene oxide synthase; DiHODE, dihydroxy-octadecadienoic acid; DOX, dioxygenase; EAS, epoxy alcohol synthase; LDS, linoleate diol synthase.

The active sites of 5,8-LDSs showed that αF and αI with the FxxxF (SRS2) and NQxQ (SRS4) sequences were positioned in the vicinity of the heme cofactor of P450 BM3 in superposition (Fig. 6*A*). AF2 models even of very high confidence may assign rotamer configurations incorrectly (73, 77), which might explain the two conformations of Gln890 and its homolog of the two 5,8-LDSs. The corresponding five residues of SRS2 and SRS4 of two 10*R*-EASs aligned with almost identical configurations (Fig. 6*B*).

Site-directed mutagenesis of SRS2 and SRS4 altered catalysis. The Asn residues at SRS4 of two 5,8-LDSs (Q0CQP2; B0Y6R2) were not essential as the N878L and N887Q replacements shifted the hydroxylation of 8*R*-HPODE partly from C5 to C6 and C7 (110). Replacement of the next residue (Q879L) did not alter catalysis. Replacements of Q881 and Q890 (Fig. 6*A*) with Cα atoms over 10 Å from the heme iron, partly retained the 5,8-LDS activity but altered the profile of oxidation products (110). Three replacements (Q881D, Q881E, and Q890E) formed 8,11-DiHODE and replacements of Q881 with of E, D, and N also produced an epoxy alcohol, 10-hydroxy-8(9)epoxy-12*Z*-octadecenoic acid (∼80% of the *threo* isomer by Q881E) (111). The N938L replacement of 7,8-LDS (Q9UUS2) virtually abolished catalysis, but the N938D and N938Q replacements only reduced the activity. N886D replacement of 6,8-LDS (S7ZN32) abrogated the LDS activity in contrast to 7,8-LDS (81). The Q889A and Q889E replacements of 6,8-LDS formed 8,11-DiHODE as the main product in analogy to Q881D of 5,8-LDS. F794E, F794L, and F794Y replacements shifted oxygenation from C6 to C8 with biosynthesis of 7,8-DiHODE as the main product but it was formed only in small amounts (81).

### 10*R*-EAS and the SRS

10*R*-EAS converts 10*R*-HPODE to an epoxide (12*S*(13*R*)-epoxy-10*R*-hydroxy-8*E*-octadecenoic acid) by intramolecular epoxidation in analogy with the intramolecular hydroxylation by 8*R*-LDS (*cf.* Fig. 1*B*). This differs from the biosynthesis of epoxy alcohols by homolytic cleavage of hydroperoxides (7, 39). 10*R*-EAS also transformed the 10*R*-hydroperoxide of ALA, but 10*S*- and 9-HPODE were not substrates (100). 13*R*- and 13*S*-HPODE were converted to *threo*-12(13)-epoxy-11-hydroxy-9*Z*-octadecenoic acids.

The SRS of 10*R*-EAS, 5,8-LDS, and the other 8*R*-LDS revealed many conserved residues, and the residues of SRS4 differed in only a few positions (Table 3). Superposition of the Phe821 and Phe835 residues of 10*R*-EAS with the corresponding Phe residues of 5,8-LDS suggested alignment of Cα atoms and side chains in slightly different positions (*cf.* Fig. 6, *A* and *B*). These Phe residues may interact with the double bonds for positioning of 8*R*- and 10*R*-HPODE at the metal center (34). The SRS4 of 10*R*-EAS also contained the Asn residue of 8*R*-LDS at the axial distal position of the heme iron. The N965V replacement of 10*R*-EAS only reduced the catalytic activity (100). The Asn residue was thus dispensable. In contrast to 5,8-LDS, the Q968L replacement of 10*R*-EAS had no apparent effect (100).

In conclusion, Asn residues at the distal axial position of the heme iron may facilitate heterolytic cleavage of hydroperoxides but they can be dispensable in both 8*R*-LDS and 10*R*-EAS. Asn residues in this position of PGIS and plant AOS may facilitate homolytic scission of endoperoxides and hydroperoxides (*vide infra*).

## 8- and 9-AOS and the AF2 models

The reaction mechanism of 8*R*-AOS with formation of 8*R*(9)-epoxy-9,12*Z*-octadecadienoic acid (with two nonconjugated double bonds) is shown in Figure 1*C*. 9*R*-AOS transformed 9*R*-HPODE in the same way to 9*R*(10)-epoxy-10,12*Z*-octadecadienoic acid, but with two conjugated double bonds in analogy with allene oxides produced by plants (4, 39).

### 8*R*- and 8*S*-AOS and the SRS

8*R*- and 8*S*-AOS transformed the 8-hydroperoxides of OA, LA, and ALA to unstable allene oxides, which were detected after hydrolysis to α-ketols (68). Their substrate specificities differed. 8*R*-AOS transformed 9*R*-HPODE to epoxy alcohols and not to an allene oxide. 8*S*-AOS isomerized 9*S*-HPODE to an allene oxide and to epoxy alcohols in a ratio of ∼1:2, but 8*R*-HPODE was a poor substrate (68).

SRS2 with two Phe residues and SRS4 with the (T/I)QxQ sequences of 8*R*- and 8*S*-AOS in are illustrated by superposition in Figure 6*C*. The Asn residue of SRS4 of 8*R*-LDS was thus replaced in 8*R*- and 8*S*-AOS by Thr and Ile residues, respectively, but the other four residues were retained. The lining residues of 8*R*- and 8*S*-AOS only varied at a small number of positions (Table 3). The SRS-αE of 8- and 9-AOS had no counterparts in 8*R*-LDS and 10*R*-EAS (34). The positions of Cα atoms and side chains of three identical residues of 8*R*- and 8*S*-AOS differed slightly (*cf.* F770, F774, and Q864 of 8*R*-AOS; Fig. 6*C*). These residues have not yet been studied by mutagenesis, but they may position the 8*R* and 8*S* stereoisomers of 8-HPODE at the metal center.

### 9*R*- and 9*S*-AOS and the SRS

9*S*-AOS isomerized the *S* stereoisomers of the 9-hydroperoxides of OA, LA, and ALA and the 11-hydroperoxide of 20:2*n*-6 to allene oxides (and identified as α-ketols), but the corresponding *R* stereoisomers were not isomerized (67, 91, 111). 9*R*-AOS isomerized the 9*R*-hydroperoxides of OA and LA and also 9*S*-HPODE to allene oxides but not the 9*R*- and 11*R*-hydroperoxides of ALA and 20:2*n*-6, respectively (64, 65, 67, 95, 111). This suggested that the active site of 9*R*-AOS could be too small to accommodate these two hydroperoxides, which was also appeared to be supported by the AF2 model of the active sites.

SRS2 and SRS4 of 9*S*- and 9*R*-AOS differed only at a few positions (Table 3). Phe867 of 9*R*-AOS might restrict the volume of the substrate cavity in comparison with Val861 of 9*S*-AOS (Fig. 6*D*). This could explain their different substrate specificities. 9-AOS differed markedly from 8-AOS in the SRS2, SRS4, and SRS5 (Table 3). The SRS2 of 9-AOS showed that the FxxxF sequences of 8-AOS, 8*R*-LDS, and 10*R*-EAS were replaced by the RxxxV/F sequences. The SRS4 of 9*S*- and 9*R*-DOX with the (V/A)PxT and TPxG sequences, respectively, also differed from 8*R*-LDS and 10*R*-EAS (Fig. 6, *A*, *B*, and *D*) (67).

In conclusion, the active sites of 8*R*/8*S*- and 9*R*/9*S*-AOS differ markedly, except for the polar (Thr) and nonpolar residues of 8*R/9R*- and 8*S*/9*S*-AOS enzymes, respectively, at the distal axial position of the heme iron. The Asn residue of plant AOS in this position was apparently not required.

## Comparison with one plant and three human CYP prototypes

A brief comparison of the SRS4 of the CYP fusion partners with SRS4 of plant AOS (Cyp74A), PGIS (CYP8A1), and the AF2 models of TXS (CYP5A1, pLDDT 91.4) and PGH_2_ 19*R*-hydroxylase (CYP4F8, 91.1) might illustrate the catalytic differences.

### PGH_2_ 19*R*-hydroxylase

The catalytic groove (SRS4 with TF*m*FGG*hd***T^332^**T) of PGH_2_ 19*R*-hydroxylase of seminal vesicles contains three lining Thr residues (including one of an acid–alcohol pair) with Thr332 (in bold) facing the heme iron (34, 112). Thr residues have been proposed to facilitate heterolytic cleavage of O_2._ by proton delivery to the heme-bound dioxygen (Fe^III^O_2_) of conventional CYP (33, 113, 114, 115). CYP4F8 might belong to this group. 8*R*-LDS and 10*R*-EAS mimic the oxygen-rebound mechanism of 19*R*-hydroxylase (*cf.* Fig. 1*B*). 19*R*-hydroxylase abstracts the 19*R* hydrogen of PGH_2_ and then rarely also a hydrogen at C18 or C20 to produce the 18,19- and 19,20-dehydro-PG compounds of human seminal fluid (116, 117). This catalytic cycle with the conversion of O_2_ to water can be considered an AOS-related transformation in the last of the two reactions (*cf.* Fig. 2*C*) (118).

### AOS, PGIS, and TXS

AOSs of fungi and plants form allene oxides by homolytic cleavage of HPODE (*cf.* Fig. 1*C*). Plant AOS can support this scission by Asn321 of the SRS4 sequence (FATcF**N^321^**TwGGMKI), positioned close to the heme iron, and as indicated by hydrogen bonds between the amide side chain and the hydroxyl group in the 3D structure of the AOS:13*S*-HODE complex (119). The Asn321Gln mutant also formed the allene oxide, but in small amounts (119). The Asn residue was therefore not indispensable, but replacements with other residues than Gln were not investigated.

PGH_2_ is isomerized by homolytic scission of the endoperoxide to form PGI_2_ and TxA_2_ *via* an alkoxyl radical at C9 and C11, respectively, and rearrangements (32). Asn287 of the SRS4 sequence (LQlWATQG**N^287^**MgPA), close to the heme iron of PGIS, may facilitate the scission by direct or water-mediated hydrogen interactions (120). The AF2 model of TXS aligned in superposition with Ile345 of SRS4 sequence (QafIFlIAGYE**I^345^**ITNT) at the position of Asn287. Homolytic cleavage of an endoperoxide (PGH_2_) can thus occur with both an amide and a nonpolar residue at the metal center. 8*S*- and 9*S*-AOS also contained a nonpolar residue in the distal axial position of heme iron, but 8*R*- and 9*R*-AOS contained a polar (Thr) residue (Fig. 6, *C* and *D*). Thr residues of SRS4 may support proton delivery to Fe^III^O_2_ of conventional CYP (114, 115), but 8*R*- and 9*R*-AOS and the other CYP fusion partners are self-sufficient as far known.

In summary, TXS and 8*R*/8*S*- and 9*R*/9*S*-AOS illustrate that the homolytic scission of oxygen–oxygen bonds might be facilitated without the axial Asn residue of heme iron of human PGIS and plant AOS. An axial Asn residue may also facilitate the heterolytic scission of 8*R*-HPODE by 8*R*-LDS. The homolytic or heterolytic scission of oxygen–oxygen bonds might therefore also be controlled by steric factors and other mechanisms.

## Discussion

AF2 models of several eukaryotic and one prokaryotic DOX of the peroxidase-COX superfamily were reviewed. It was reassuring that the AF models and the experimental 3D structures of five COXs aligned the Cα atoms with rmsd < 0.5 Å (Table 1). The catalytic parts of the DOXs and their CYP companions were mostly predicted with very high confidence. Studies on the reaction mechanisms and physiological functions may therefore add more significant information than improvements of the 3D structures with two crucial exceptions. The AF2 models do not reveal the position of fatty acids and hydroperoxides in the active sites. The 3D structures of the DOX:LA and the CYP:HPODE enzyme complexes will solidify our knowledge of the catalytic mechanisms in the same way as the pioneering 3D structures of the enzyme:substrate complexes of COX, PGIS, and plant AOS (2, 63, 119, 120, 121).

Smith and co-workers analyzed the SRS of the COX-1:AA complex with site-directed mutagenesis and identified a series of critical amino acid residues for catalysis (2, 40, 41, 42, 43). This was extended to residues in control of the cyclic reaction and the oxidation of C15 by COX-1 and COX-2 as briefly discussed above (3, 87, 88). The transformation of AA to PGH_2_ is a complex reaction and supported by steric factors. SRS of the central motifs of COX and DOX illustrate the similarities in the vicinity of the central and catalytic Tyr residues (Table 2). The V349L replacement of COX augmented the biosynthesis 11*S*-, 15*R*-, and 15*S*-HETE to 24% of total products (41, 43). 8*R*- and 10*R*-DOX have V330 and L384, respectively, in this position and a second hydrophobic pair (L334 and V388) at fourth position further down (Table 2; Fig. 5*E*). Site-directed mutagenesis of these positions, Val to Leu and *vice versa*, altered O_2_ insertion to C10 by 8*R*-DOX and to C8 by 10*R*-DOX (101). The terminal motif also controls product formation. The inhibition of COX by acetylation of Ser530 by aspirin with formation of 15*R*-HETE is the best-known example (87, 89). Two replacements of the terminal motif of 9*R*-DOX also altered the biosynthesis to 9*S*-HPODE (>90%) (Fig. 4*C*) (95). SRS of the five fungal DOXs illustrate key positions in known or hypothetical control of hydrogen abstraction and insertion of O_2_ and they also describe the evolution of the catalytic sites by alterations of the central and terminal motifs.

COX with its unique catalytic properties, cyanobacterial 10*S*-DOX, and fungal DOXs may have evolved from an ancient bacterial ancestor (54), which was gradually transformed into three different directions. The LDS subfamily may illustrate one of these developments from a hypothetical precursor, pre-DOX (93, 94). The latter may have bound LA in carboxylate-in orientation and sequentially evolved into 8*R*-DOX, 8*S*-DOX, and 10*R*-DOX by altered positions for insertion of O_2_ (Table 2). The pre-DOX may also have bound LA in carboxylate-out orientation and sequentially evolved into 9*S*- and 9*R*-DOX. The five DOXs also align in superposition with modest deviations of the Cα atoms of the catalytic domains (rmsd 0.34–0.60 Å). Their structures differ from COX and 10*S*-DOX by their unique MBD. Nevertheless, the α-helices of these DOX align in superposition with COX and 10*S*-DOX with rmsd between 1.2 and 1.6 Å (35).

The cyanobacterial 10*S*-DOX appeared to be closer related to COX than to the fungal DOXs based on the alignment of three conserved α-helices of the MBDs and the α-helices of the catalytic domains with only modest deviations of Cα fold (rmsd 1.2 Å) (*cf.* Fig. 2, *C* and *D*) (34, 55). 10*S*-DOX also shows similarities with the eukaryotic DOXs in the terminal motif of the SRS (Table 2) and in the distal face of the heme cavities, which were not shared with COX (Fig. 2*E*). This indicates a multifaceted evolution of COX, 10*S*-DOX, and LDS.

The CYP fusion companions of the DOXs with the HxCxG motif in the Cys pocket might originate from an ancient bacterial ancestor of the CYP superfamily, which was once fused to a member of the peroxidase-COX superfamily (54, 122). The α-helical structure and the β-strands of 5,8-LDS, 8*R*- and 9*R*-AOS were mainly retained and they aligned with only a modest deviation of the Cα atom fold (rmsd ∼1.2–1.6 Å) (34). The CYP fusion companions apparently adjusted the active sites as the DOXs evolved from biosynthesis of one HPODE to another, which might have failed in 10*R*-DOX-1 (62, 123).

The homolytic isomerization of the endoperoxide of PGH_2_ by PGIS and TXS involves complex rearrangements to form PGI_2_ and TxA_2_ (32). Less is known about the control of the scission of the endoperoxide. Human PGIS contains Asn287 close to the metal center in analogy with Asn residues of 8*R*-LDS, 10*R*-EAS, and plant AOS. TXS, 8*S*-, and 9*S*-AOS have nonpolar amino acid residues in this position, and 8*R*- and 9*R*-DOX a polar (Thr) residue. These enzymes might facilitate cleavage of oxygen–oxygen bonds by different mechanisms but the presentation of the hydroperoxide group at the metal center by steric factors could be a critical factor.

Five conserved residues in the AF2 models of SRS2 and SRS4 appeared to stabilize 8*R*- and 10*R*-HPODE in correct positions for oxidation by 8*R*-LDS and 10*R*-EAS (Table 3; Fig. 6, *A* and *B*). Site-directed mutagenesis of these residues of 8*R*-LDS often shifted the position of the intramolecular hydroxylation and usually reduced catalysis. Asn878 (SRS4) was not required for 5,8-LDS catalysis as the Asn878Leu replacement produced 5,8-, 6,8-, and 7,8-DIHODE (110). Four of the five key residues of SRS2 and SRS4 of 8*R*-LDS were conserved in 8-AOS, but the Asn residue was replaced by Thr and Ile residues of 8*R*- and 8*S*-AOS, respectively (Fig. 6*C*). It raises an obvious question: Will it be possible to convert 8-AOS to any of the four 8*R*-LDS by replacement of Thr or Ile with Asn or *vice versa*? These experiments could help to elucidate the mechanism of homo- and heterolytic scission of 8-HPODE.

In summary, the AF2 models of DOXs and their CYP companions have supplemented the catalytic properties with structural and mechanistic information in a pioneering manner. Amino acids in conserved places of the SRS can direct the position and orientation of molecular oxygen insertion at carbon chains and also alter the intermolecular transformation of hydroperoxy fatty acids by the CYP fusion partners. It has now been possible to analyze and compare the protein fold, the SRS, the catalytic properties, and the evolutionary relationships with COX and bacterial DOX in greater detail than ever before. The AF2 models also revealed numerous amino acids as targets for consequential investigations.

## Conflict of interest

The authors declare that they have no conflicts of interest with the contents of this article.
